# Acceptability of a novel suicide prevention psychological therapy for people who experience non‐affective psychosis

**DOI:** 10.1111/papt.12456

**Published:** 2023-03-01

**Authors:** Kamelia Harris, Patricia A. Gooding, Yvonne Awenat, Gillian Haddock, Leanne Cook, Charlotte Huggett, Steven Jones, Fiona Lobban, Ellen Peeney, Daniel Pratt, Sarah Peters

**Affiliations:** ^1^ Division of Psychology and Mental Health, School of Health Sciences, Faculty of Biology, Medicine and Health University of Manchester Manchester UK; ^2^ Manchester Academic Health Sciences Centre (MAHSC) Manchester UK; ^3^ Greater Manchester Mental Health NHS Foundation Trust Manchester UK; ^4^ Lancashire and South Cumbria NHS Foundation Trust Lancashire UK; ^5^ Department of Health Research Lancaster University Lancaster UK; ^6^ Manchester Centre for Health Psychology, Division of Psychology and Mental Health, School of Health Sciences, Faculty of Biological, Medical and Health Sciences University of Manchester Manchester UK

**Keywords:** acceptability, cognitive behavioural therapy, intervention, psychological therapy, psychosis, qualitative study, suicidality, suicide

## Abstract

**Objectives:**

Suicide is a leading cause of death worldwide. People experiencing psychosis are at increased risk of death by suicide. Talking therapies can alleviate suicidal thoughts, plans, and attempts. Therapies need to also be acceptable to recipients. The aim of this study was to investigate the views on psychological therapy for people experiencing psychosis and suicidality using the Theoretical Framework of Acceptability.

**Design:**

Qualitative interview study.

**Methods:**

Participants were recruited from a randomised controlled trial comparing suicide prevention psychological therapy with treatment as usual. Individuals had a diagnosis of non‐affective psychosis and experience of suicidal thoughts, plans and/or attempts. To assess the acceptability of the therapy, semi‐structured interviews were conducted with 20 participants randomised to receive therapy. Data were deductively analysed using an adaptation of the Theoretical Framework of Acceptability.

**Results:**

Interviews (*Mean* = 45 min) were conducted and audio recorded with 21 participants. Data were organised into six themes: 1. Affective attitude, 2. Burden, 3. Alliance, 4. Intervention coherence, 5. Perceived effectiveness, and 6. Self‐efficacy. There was no evidence of issues relating to domains of ethicality and opportunity costs associated with receiving therapy.

**Conclusions:**

Talking about suicide was difficult and, at times, distressing, but it was perceived to be useful for understanding experiences. To be acceptable, it is important for therapists to ensure that clients' understanding of therapy aligns with expectations of effectiveness and to invest in building strong therapeutic alliances. Future research will benefit from examining therapists' experiences of delivering therapy through different modes (e.g. online, telephone).

**Practitioner Points**

1. Talking about suicidal experiences during the intervention was described as effortful, difficult, at times, distressing and beneficial for understanding mental health problems and suicidal experiences.
2. It is important to consider individuals' preference for mode of therapy (e.g., telephone, face‐to‐face, online) as this may influence therapy attendance and engagement.
3. Therapists need to ensure that clients' understanding of therapy aims is clear and aligns with their expectations of effectiveness and to tailor the therapy to an individual's needs.
4. The Theoretical Framework of Acceptability used in this study could be usefully expanded by incorporating therapists' experiences and perceptions of delivering therapy.

## INTRODUCTION

Over 700,000 people die by suicide every year globally (World Health Organization, 2021). In 2019, there were 5691 deaths by suicide in the UK (Office for National Statistics, 2020). People with mental health problems are most vulnerable to suicidal experiences (Hawton & van Heeringen, 2009; O'Connor et al., 2021). Individuals experiencing psychosis are at a particularly high risk of suicide death (Hawton et al., 2005; Saha et al., 2007; Tarrier et al., 2014) with a 13‐fold risk of suicide compared to the general population (Too et al., 2019). A sixfold increase in suicide deaths in people with diagnoses on the schizophrenia spectrum, including non‐affective psychosis, compared to people experiencing affective mental health problems was reported in a meta‐analysis (Chapman et al., 2015). Around half of the people with psychosis think about and/or attempt suicide in their lifetime (Aydin et al., 2019; Hawton et al., 2005). Experiencing suicidal thoughts and behaviours, social isolation, and feeling hopeless, defeated, and trapped can increase the risk of suicide death (Bornheimer et al., 2020; Harris et al., 2021; Owen et al., 2015; Panagioti et al., 2011; Pinikahana et al., 2003; Stravynski & Boyer, 2001). Furthermore, living with psychosis can be immensely distressing and suicide can be perceived as a way to escape distress (Harris et al., 2020).

There is growing evidence that psychological interventions which target suicidality can reduce suicidal thoughts, acts, and urges (Hawton et al., 2016). Cognitive Behavioural Therapy (CBT) has been a recommended approach for helping people who experience suicidal thoughts and behaviours (National Institute for Health and Care Excellence, 2022). Interventions need to be effective, but also acceptable, to recipients and those delivering them (Medical Research Council, 2021; Sekhon et al., 2017). Over the last decade, CBT‐based suicide interventions have been developed and studies have confirmed the interventions' potential in terms of effectiveness and acceptability in individuals in inpatient wards (Awenat et al., 2018; Awenat, Peters, et al., 2017; Awenat, Shaw‐Núñez, et al., 2017; Haddock et al., 2019), prisons (Pratt et al., 2015), and the community (Tarrier et al., 2014). Given the elevated risk of suicide in individuals with psychosis, it is important to investigate the acceptability of interventions that specifically target suicidal experiences in the context of psychosis (Gooding et al., 2020).

The acceptability concept has been poorly operationalised within the literature (Sekhon et al., 2017; Sidani et al., 2009; Staniszewska et al., 2010), leaving researchers without a common language or means of assessing treatment acceptability. To address this, Sekhon et al. (2017) developed the Theoretical Framework of Acceptability (TFA) which defines acceptability as the extent to which a healthcare intervention is considered appropriate for individuals who receive and deliver it. Most studies using the TFA have focused on physical health problems and health behaviour change (Brookfield, 2019; Gossage‐Worrall et al., 2019; Griffin et al., 2019; Kurniawati et al., 2019; Murphy & Gardner, 2019; Nadarzynski et al., 2019; Palsola et al., 2020; Rockliffe et al., 2018). The TFA is yet to be applied to psychological interventions targeting suicidal thoughts and behaviours in the context of psychosis. Examining the acceptability of therapy for such complex experiences is important for understanding issues of accessibility, engagement, and uptake, and ultimately, for reducing suicidal experiences.

The primary aim of this study was to utilise the TFA to investigate participants' views on the acceptability of a suicide‐focused CBT for people experiencing non‐affective psychosis (i.e., CBSPp) in the context of a randomised controlled trial (RCT; Gooding et al., 2020). CBSPp is a one‐to‐one therapy offered in 24, 50‐min sessions with a therapist trained in CBT and CBSPp. It is underpinned by the general principles employed when using more generic CBT for people with psychosis. However, CBSPp aimed to ensure that the formulation and intervention focused specifically on understanding how those concepts believed to underpin suicidality as highlighted by the Schematic Appraisal Model of Suicide (SAMS, Johnson et al., 2008) were incorporated into the formulation and intervention (see Tarrier et al. (2013) for a thorough description of the CBSP approach). This study aimed to address the following research questions:

1. What were people's views and experiences of receiving CBSPp?
2. How do people's views and experiences of CBSPp map onto the TFA?

## METHODS

Semi‐structured interviews were conducted with 20 participants randomly allocated to receive CBSPp as part of a multi‐site RCT investigating the efficacy of the intervention (i.e., Cognitive AppRoaches to coMbatting Suicidality (CARMS); Gooding et al., 2020). Participants for the trial were recruited from community and inpatient settings across four NHS trusts in North‐West England and were randomised to either CBSPp plus treatment as usual (TAU) or TAU alone. Ethical approval was granted by an NHS Research Ethics Committee (17/NW/0089).

### Patient and public involvement (PPI)

An expert by experience group, self‐titled ‘the CARMers’, who comprised individuals with experiences of psychosis and suicidality was specifically involved in the design and dissemination of the project. Two members presented their experiences of being involved in the trial at a national conference. The group were consulted every two months throughout the study, inputting in the development of the topic guide, data analysis and dissemination materials (e.g. presentations, posters).

### Participants

Inclusion and exclusion criteria for the trial are detailed in the CARMS protocol (see Gooding et al., 2020) but briefly comprise: 1. Experience of non‐affective psychosis according to ICD‐10 criteria confirmed by the participant's care team; 2. Suicidal experiences in the three months prior to recruitment into the trial confirmed by the participant's care team; 3. Being under the care of an NHS mental health team at the time of consent; and 4. Ability to give informed consent (determined by a member of the participant's care team). Exclusion criteria were presence of dementia or organic brain disorder, and inability to complete assessments due to language barriers and/or partaking in another clinical trial. All participants in the study provided either written or voice‐recorded telephone informed consent prior to their participation.

Participants for this qualitative study were recruited following therapy completion as part of an RCT investigating the efficacy of a novel psychological therapy for suicidality in people experiencing non‐affective psychosis (CBSPp; Gooding et al., 2020). Participants were identified through purposive sampling to ensure maximum variance in views and experiences, such as age, ethnicity, suicidal thoughts and behaviours, and length of time being under the care of mental health teams (Kelly, 2010; Palinkas et al., 2015; Patton, 2002). All participants were under the care of a community mental health team at the time of the study. For up‐to‐date risk information, the participant's care coordinator was contacted in advance of interviews. An information sheet was sent to those interested and participants were given at least 24 hours to consider whether they would like to take part. Participants were given £10 as a ‘thank you’ for participating in the interview.

The mean age of the participants in this study was 38 years (*SD* = 13.68), 65% identified as female, 90% were White British, 5% were Asian and 5% were Mixed Race. The most common diagnosis was schizophrenia (65%), followed by unspecified nonorganic psychosis (15%), schizoaffective disorder (10%), other non‐organic psychotic disorder (5%), and persistent delusional disorder (5%). These data were collected as part of the CARMS RCT. On average, participants attended 17 therapy sessions (*SD* = 6.21; range 0–24). Of note, due to social restrictions during the COVID‐19 pandemic, therapy sessions with seven participants were conducted remotely (i.e., by telephone or online platform) with their consent.

### Data collection

Interviews were conducted by LC, YA, PM and KH between March 2018 and June 2021. A flexible topic guide was developed from previous literature and experience of designing and piloting the intervention by the research team. The topic guide was designed to examine therapy acceptability and applied to the TFA. Initial interviews were conducted face‐to‐face at either participants' homes or clinical settings (e.g. GP surgery). Following the COVID‐19 restrictions in March 2020, interviews were conducted by telephone.

Interviews were audio recorded using an encrypted device, transcribed verbatim by a professional transcription company, and checked for accuracy by the interviewers. Identifying information was removed from transcripts. The median interview was 43 min (*SD* = 10 min; *Range* = 27–63 min).

### Data analysis

**FIGURE 1:**
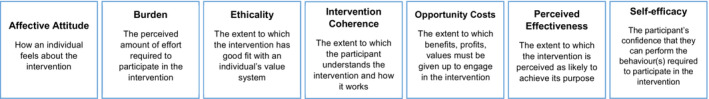
Original Theoretical Framework of Acceptability (Sekhon et al., 2017).

Data were analysed inductively, using thematic analysis (Braun & Clarke, 2006, 2021) and deductively, using the original TFA which includes seven domains, namely 1. Affective attitude, 2. Burden, 3. Ethicality, 4. Intervention coherence, 5. Opportunity costs, 6. Perceived effectiveness, and 7. Self‐efficacy (Sekhon et al., 2017; see Figure 1).

Data were organised using NVivo version 12 (QSR International Pty Ltd, 2018). The analysis involved data familiarisation through repeated reading of transcripts. These were then coded line by line in NVivo using a coding manual based on the TFA (Sekhon et al., 2017). In addition, through inductive coding of initial transcripts, a scoping review of the existing literature on the acceptability of mental health interventions, and previous studies that have used the TFA (Brookfield, 2019; Gossage‐Worrall et al., 2019; Kurniawati et al., 2019; Murphy & Gardner, 2019; Palsola et al., 2020; Rockliffe et al., 2018), the coding manual was updated to fit with the experiences of people receiving CBSPp. Analysis was driven by the domains of the TFA and the recognition that researchers are subjective and knowledge is context‐dependent (Braun & Clarke, 2021). Therefore, meetings were held with the research team where the coding of incoming data and the coding manual were discussed and further refined to ensure clarity and cohesiveness. The coding manual was then deductively applied to the full dataset by LC, KH and SP. To ensure the reliability of the coding process, 20 codes were selected, and an independent rater allocated them to the TFA themes. The per cent agreement was 80.

## RESULTS

**FIGURE 2:**
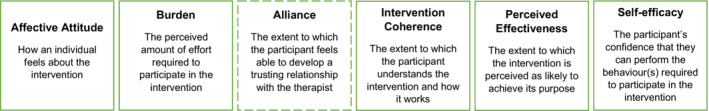
Adapted Theoretical Framework of Acceptability applied to CBSPp.

No data were found for two of the original TFA domains (i.e., *Ethicality* and *Opportunity costs*) and a new domain was created (i.e., *Alliance*), which captured the acceptability of working relationships with the therapist (see Figure 1 for the original and Figure 2 for the adapted TFA).

### Theme 1: Affective attitude

This theme describes how people felt about the CBSPp intervention. Affective attitude is characterised by any expression of thoughts and feelings about the therapy, capturing both positive and negative attitudes of talking about suicide during therapy.

#### Perceptions of therapy

The participants in this study had a positive view of CBSPp and found it useful and worthwhile. They described feeling ‘over the moon’ (ID 14) with being allocated to the therapy condition because it met a therapeutic need: ‘I was like, oh, CBT, I've heard of that. That could be useful.’ ‘Cos I've always wanted to access, like, long‐term speaking therapy.’ (ID 11). Specific aspects demonstrated a positive outlook, such as feeling supported, involved, and in control of an intervention that was tailored to individuals' needs: ‘it felt like I was being supported, it did not feel like they [the therapist] were the one in control’ (ID 08). Exploring and learning about mood changes were particularly useful for people: ‘it was just useful to see what my mood was when I started. And then each session revisiting where it was as opposed to the previous session.’ (ID 15).

Opening up about problems and having someone to talk to who listens and understands were examples of positive attitudes towards CBSPp. Although talking to someone is helpful, there can be a level of uncertainty because of the potential consequences that ensue as a result of opening up:

'To talk is very important […] But when you do not feel in control of it, you do not know what's coming out. Because I'm hearing such badness sometimes, I'm thinking, have I said any of that? […] I do not feel in control, so, I'm worried that something wrong comes out.' (ID 04).

Conversely, there were negative views of CBSPp for three reasons. First, some participants did not like talking about suicidal experiences. This resulted in low expectations of the effectiveness of CBSPp. For others, talking about such experiences was extremely overwhelming and distressing:

we dug into the past […] but it was too much […] I'd had like three nights with nightmares of things that had happened, or things I could have done different. (ID 04).

Participants also worried about being judged and nervous about meeting someone new. Despite those fears, people reported being willing to go through with the therapy because of the potential to receive help. Second, there were concerns expressed about managing well‐being after the end of therapy but participants understood that therapy was going to end at a specified time:

I was a little bit worried thinking how I would cope after the therapy, but I was okay […]. The therapist told me what happens after that, like how I can cope in case I'm not feeling too good. (ID 21).

The third type of negative view was that the number of therapy sessions received whilst in the trial was insufficient. Furthermore, some participants felt that therapy ended abruptly and at a time when they had more issues to discuss: ‘I've learnt from hearing voices, it's come from childhood experiences. I wish I could have explored it a bit more, but we did not have time.’ (ID 01). However, this was offset by others who felt like therapy ended at the right time: 'It felt it was alright – it did not feel like I was just sort of thrown, like, right here you go and that's it now, […] it felt that I was ready…' (ID 13). Several participants did not have any expectations and were open to trying a new intervention regardless: 'I had no expectations besides the fact that, oh, they are trying something new […] let us see how it goes.' (ID 21).

#### Talking about suicidal experiences during therapy

Most participants welcomed the idea of opening up about their suicidal experience during therapy and felt that it was worthwhile and helped reduce suicidal experiences: ‘I think it's very, very helpful, um, certainly if they can prevent people committing suicide’ (ID 019). Talking about suicide was challenging but a worthwhile endeavour which ultimately had a positive effect on suicidal experiences and mental wellbeing: ‘Having to sit there and go through everything and relive everything, it was the hardest thing. But I did it. […] I thought I achieved a lot doing that.’ (ID 05).

Some people had concerns about the consequences of talking about suicidality: ‘I just panic that I'll get locked up [laughs]. That's a big fear because I was in hospital such a long time.’ (ID 10). Other concerns related to anticipated exacerbation of distress and suicidal experiences: ‘Well, I know when I talk about suicide it can cause me to feel a bit suicidal, that's just the way it is.’ (ID 19). Therefore, it was perceived as scary and not something they talked about. This was due to the potential need to want to protect significant others from this burden and the emotional impact of discussing traumatic and distressing experiences which sometimes led to low mood. It is important to note that these were anticipated and transient, rather than experienced, effects of therapy, as one participant explained: ‘It was transient, it was fleeting, it just passed, and I had negative moods when I spoke about suicide, but it did not last.’ (ID 19).

Participants liked that the therapy focused on suicidality as this was a pertinent problem that they wanted to address, and reported a decrease in self‐harm and suicidal experiences during and in the months following CBSPp: ‘since the therapy, I'm not saying I've not self‐harmed but, it's not been as much.’ (ID 18). Overall, participants' experiences of CBSPp were positive, despite the challenges associated with talking about suicidality: ‘everybody I spoke to was very kind, very friendly, non‐judgemental. […] it's really lovely people to work with.’ (ID 08).

### Theme 2: Burden

Examples of burden included emotional impact of therapy, amount of homework and associated paperwork, and barriers to therapy attendance, such as inability to travel and difficulty focusing for a long time.

#### Effort required to participate in CBSPp

Talking about suicidal experiences during therapy was described as effortful and, at times, distressing. This experience was counterbalanced by the perceived benefits of the intervention for participants' well‐being. Establishing a connection with the therapist counteracted the emotional burden:

I had to […] challenge myself into doing something because I knew that you had to go through difficult stuff to get through the other end. But it's like, um, having the therapy sometimes made me feel worse. And there were a couple of times when I did not want to do it, but I pushed myself through that barrier and completed it. But I suppose if there had not been somebody I could connect with, I think I'd have given up. (ID 18).

A technical burden related to the paperwork and homework tasks (e.g., daily activity diary) that participants were invited to do during therapy. Some participants found this to be burdensome and that it affected their confidence and ability to use resources:

I would not really turn to them [therapy materials] in an emergency […] because there were so many papers, and there were so many flow charts, and I'm not used to flow charts. […] that was a bit of an obstacle. (ID 06).

#### Impact of different modes of therapy delivery

At the start of the pandemic, the CBSPp therapy team had to adapt to effective ways of delivering sessions over the telephone and/or online through live voice calls. For some people, receiving a telephone intervention was burdensome: ‘I was a bit scared at first because […] I just could not get talking on the phone, talking on the phone I were really – I do not know, weird.’ (ID 14). However, many participants preferred telephone therapy for practical reasons, such as not having to get dressed and travel to an appointment. Others felt more able to talk about suicidality over the phone:

I think it's easier on the phone because if I'm face‐to‐face with someone and they're asking about suicide, how I plan it out and how I just feel like throwing myself in front of a train when I'm that low, and I can't really say that to someone face‐to‐face. I feel embarrassed. It's not easy to open up, whereas on the phone it's like talking to yourself. (ID 17).

Having shorter sessions and receiving therapy at home, by telephone or online were tangible ways to relieve burden, though some participants did not have a preference for a particular mode of therapy:

I didn't mind, to be honest, like, I don't think I would mind either. If I had to pick, it didn't really make loads of difference to me. (ID 13).

### Theme 3: Alliance

Building trust in the client‐therapist relationship was an important aspect of therapy which had implications for establishing a therapeutic alliance and increasing acceptability. Most participants felt that they established a trusting relationship with their therapist which allowed them to discuss sensitive issues safely:

the safety is that you're telling someone who has this knowledge. And someone who has this kind of understanding and have been around people like me. (ID 08).

However, some found this relationship difficult to develop at the start of therapy. This was for two reasons, firstly, due to participants' mistrust and anxiety about meeting new people. The second related to the challenges of talking about sensitive issues, such as suicidality, over the phone:

it took us a couple of months for me to be so forthcoming and that's because I was just a bit uncomfortable at first with the over the phone, cos it was like, I couldn't wrap my head around that you don't know who the person is on the other end. (ID 20).

Having someone who was friendly and easy to talk to facilitated building trust. Conversely, building a trusting relationship was difficult for one participant who did not feel listened to by their therapist. This was in the context of the therapist asking the participant to think of three nice things to do the following week, but the participant felt unable to do that, which made them not trust the therapist:

And then he [the therapist] said, ‘Can we do three nice things next week or can you think of three?’, and I just felt like, ‘boom’, my whole world fell apart. […] I thought I trusted him and then I couldn't trust him anymore, and that was it, that was the end. (ID 03).

An important aspect of therapy was having a mutual understanding that client and therapist can sometimes disagree about therapeutic approaches and accepting it as part of the therapeutic relationship with this leading to therapy adapting to client's preference: ‘And just an acceptance that we did not agree on this and that was it really. There were no arguments about it.’ (ID 06).

### Theme 4: Intervention coherence

Participants reported feeling that CBSPp could work as a standalone therapy and were clear about its focus on both suicidal experiences and psychosis:

I'd say it was very standalone […] but that's because I've never actually had a therapy based on the fact that I'm suicidal and that I hear voices and they're two things that I would say have been my biggest struggle. (ID 20).

Participants understood the purpose of the between‐session tasks and liked the therapy resources. Some aspects that participants worked on in sessions included focusing on positive memories and problem‐solving. In addition to those techniques, participants were provided with a staying well plan towards the end of therapy. Although most thought it contained useful information, some participants were unsure how to utilise it:

[The therapist] gave me a lot of pieces of paper with flow charts and everything. And a work plan and everything, but […] I don't notice it every day–, but maybe I should do, I don't know. (ID 06).

Of note, one participant appeared to have had a different understanding of the purpose of CBSPp which resulted in great disappointment. They had sought help with obtaining justice for personal issues, which did not directly fit with the intervention's focus on suicidality:

to me it was a wet flannel. It was going through the old thing over again after all these years, and then I thought I'll trust him [the therapist] and it all fell through, and it devastated me. (ID 03).

Four participants thought that their experiences of suicide did not warrant an intervention targeting suicidality and felt they did not fit the intervention inclusion criteria because their suicidal experiences were not severe enough. This could have been a result of varied understanding of what constitutes ‘suicidal experiences’:

I didn't really feel that I was perhaps the right person to be receiving this. […] I would kind of feel a bit of a fraud. […] I don't think I'm really going to commit suicide or even really think about it that much. (ID 06).

Despite this belief, three participants found the focus of CBSPp on suicidality and psychosis useful: ‘It did help me to […] realise probably why I was, kind of, thinking those thoughts and why I found them so distressing.’ (ID 15).

### Theme 5: Perceived effectiveness

This theme describes the extent to which the intervention was perceived as being effective in reducing suicidal experiences. It includes examples of aspects that participants found helpful and effective and/or unhelpful and ineffective.

#### Anticipated and experienced effectiveness

The CBSPp's effectiveness related to anticipated and experienced improvement in three domains, namely mental health experiences, social interactions and general well‐being. In relation to mental health experiences, participants reported a reduction in psychological distress and suicidality: ‘I did not have a panic attack, and […] did not have a self‐harm episode.’ (ID 08). Not only did people notice a reduction in suicidal thoughts and behaviours, but they also noticed a positive change in the ways they perceived and coped with these experiences: ‘when I feel suicidal, I feel more positive about the outcome. I do not feel as though I'm going to act on them [the suicidal thoughts].’ (ID 01). Conversely, one participant perceived little impact upon managing symptoms after CBSPp: ‘I have a good understanding of what causes my mental health but no real idea on how to like control or maintain my symptoms.’ (ID 20).

In relation to social interactions, people identified improvements in social activities, building connections with others, and feeling ‘more relaxed around people’ (ID 21). These changes were noticed by significant others: ‘And just everyone, like family and friends have kind of obviously noticed a massive difference in kind of how I am since I've had the therapy.’ (ID 15). An important benefit of therapy was feeling more able to engage with family and friends and talk to them about problems:

Talking to my partner, I like talking to my mum. […] get to talk about whatever it is that's bothering me, or making me feel this way, or what set me off and they'll try and like rationalise it and help me get through it. (ID 17).

In terms of well‐being, a better understanding of self and suicidal experiences was highlighted as a positive outcome of therapy. Additionally, some people reported that therapy had made them realise that they had reasons to live which, in turn, made them feel more in control of their voices and self‐harm urges:

even though I have voices in my head, it's knowing that I'm in control because nobody can hurt me, only me. […] talking about the times that I've actually self‐harmed and stuff helps me realise that I don't actually have to self‐harm. (ID 18).

These changes in well‐being had a lasting impact on people's lives: ‘I think it just feels like it's embedded now, where I do not really need to kind of really work hard to use it.’ (ID 15). Conversely, one participant reported that CBSPp only helped in the short term: ‘the therapy really helped me. […] but it does not help forever.’ (ID 05). It was considered important to continue to apply the knowledge and skills learned in therapy to experience the positive effects of the intervention in the long term: ‘I thought to myself that it's in my best interests to take this seriously and to make an effort.’ (ID 21). Four participants were open‐minded about the potential effects of therapy, did not know what to expect from therapy or had no expectations at all: ‘I had no idea really, I had no preconceived ideas.’ (ID 19).

### Theme 6: Self‐efficacy

This theme describes participants' perceived confidence to complete in‐session and between‐session tasks and implement the techniques learned during therapy in their lives. It specifically elicits how able participants felt to engage with and follow the intervention and whether there were any therapy resources that they felt able or unable to utilise independently in the long term.

#### Confidence in completing tasks

Participants had mixed attitudes towards the purpose of tasks but, nevertheless, attempted to complete them and found them useful. One participant could not complete a positive memory task because they did not have many positive memories to draw upon: ‘I wasn't having that many positive memories. The only ones that I was having was when I go around for tea to my mum's on a Sunday. […] I'm not just really into doing stuff like that.’ (ID 12). Overall, participants found the tasks useful but sometimes difficult to complete: ‘a lot of it is useful, but then I think sometimes when you are in quite a bad place […] it can be hard to get that kind of motivation to fill in diaries.’ (ID 15).

#### Confidence in utilising therapy resources post‐intervention

The techniques practised during therapy were perceived to be useful and effective by most participants in managing distress and the impact of suicidal experiences on well‐being. Some of these techniques involved practical advice on how to manage self‐harm (e.g. ‘bite on a chilli’ (ID 18)) or talking to people and trying to remember positive experiences. The perceived effectiveness of these techniques varied:

it's hard sometimes cos, when you're feeling that low, it's not like you think, oh yeah, I'll do this. But sometimes I […] talk to my partner and they instantly remind me of all the good things and bring me out of it a little bit. (ID 17).

Other examples of using resources learned in therapy included activities, such as going for a walk, listening to music, breathing and meditation, thinking about or writing down positive memories, and recording daily activities. These techniques were also helpful in particularly difficult times:

When it's so black you don't always see the moment [of joy], you're not looking for it, cause you're buried in the black. And you're not looking for that little light. But when someone says to you, “Look a bit deeper and just look for that little speck,” and you learn how to do it. It does help. (ID 04).

Conversely, a proportion of the participants reported that they had not revisited any of the resources because they did not feel they needed to or were concerned that they may make them think about suicide or worsen their psychosis symptoms.

## DISCUSSION

This study aimed to address two research questions relating to people's experiences of CBSPp and how their experiences mapped onto the Theoretical Framework of Acceptability (TFA; Sekhon et al., 2017). CBSPp was perceived as being acceptable, useful and beneficial for well‐being and understanding experiences of suicidality and psychosis. There are three key findings related to CBSPp's acceptability.

First, talking about suicidal experiences during therapy was unequivocally difficult and emotionally challenging, which corroborates the findings of previous research in the field (Harris et al., 2019; Littlewood et al., 2019; Peters et al., 2022; Richards et al., 2019). There seemed to be a ‘leap of faith’ in some participant reports where they had to overcome initial concerns and place their trust in the therapeutic process. It is important to draw attention to the contrast between participants' apprehensions about the emotional impact of therapy and the reality of their experience of attending sessions. As illustrated in the Results, participants perceived a reduction in suicidal experiences and success in building a therapeutic relationship despite initial concerns. Therefore, it is important to clearly explain to potential recipients the likely contrast between the anticipated and experienced impact of receiving the intervention when considering if they should commence therapy.

Second, the homework tasks and paperwork involved in participating in therapy were experienced as burdensome by some participants. Homework tasks are arguably an essential practice in CBT but can be accompanied with certain challenges, such as perceived inability to complete the task due to concerns about its content or difficulty, practical obstacles, or not having a clear understanding of the purpose of the task, which can impact the therapy outcome (Helbig & Fehm, 2004; Kazantzis & Miller, 2022). This suggests a need to explore problems with homework task completion with clients and modify the tasks to alleviate clients' concerns, if necessary. Negative experiences relating to homework completion in our study were negated by the usefulness of CBSPp for understanding self and developing coping skills and strategies which had a positive impact on well‐being. People discussed the value of taking part in the intervention which was a balance of perceived burden and experienced effectiveness. The burden was perceived to lessen as useful therapeutic benefits were becoming more apparent to participants. As reported in the Results, there was a notion that individuals needed to do the hard work that comes with the initial stages of the therapy and continue to apply the learned skills in order to experience the benefits associated with CBSPp in the longer term.

Third, four participants felt like they were not ‘suicidal enough’ to have CBSPp. Having an unclear understanding of the rationale and purposes of therapy can have a potentially negative impact on clients' experiences of the intervention (Brooks et al., 2021; Crawford et al., 2016). Therapists and professionals referring clients for CBSPp need to highlight that this type of intervention focuses on various suicidal experiences, such as thoughts, urges, plans, attempts and self‐harm with and/or without wish to die, in the presence or the absence of a suicidal crisis. This may help ensure that no individuals are omitted and feel that the intervention is inapplicable to their experiences, and also raises a question about who is most likely to benefit from the intervention and when.

The original TFA (Sekhon et al., 2017) fitted with the acceptability data obtained in this study with two exceptions. There was no evidence of costs associated with taking part in the intervention (i.e. Theme 5 in the original TFA). This theme describes benefits that were given up (e.g., opportunities for other therapeutic activities), and any financial (e.g., travel, public transport, parking) and time‐related costs to receiving an intervention. It is important to note that the nature of the intervention being delivered in a research setting may have resulted in no incurred costs or lost opportunities. Treatment as usual in this study did not usually include therapy. Therefore, people reported a benefit of taking part in the intervention, rather than loss of opportunities.

The second exception was the newly developed alliance theme (Theme 3 in the adapted TFA) which reflected the importance of building trust in the client‐therapist relationship. This theme is specific to talking interventions as it has implications for establishing a therapeutic alliance in ensuring intervention acceptability.

### Limitations and strengths

This study has at least two limitations which need to be considered. First, participants were recruited from an RCT investigating the efficacy of a novel psychological therapy for suicidality and psychosis. Participants in such a trial are likely to indicate some acceptability of the concept of the intervention or, at least, a willingness to engage with the intervention and motivation to complete assessments and interviews as part of the project. As such, the data collected may hold a positive bias (Borkovec & Sibrava, 2005; Button & Munafo, 2015). This has implications for the TFA in relation to the opportunity costs and ethicality domains which were not present in the study. For example, those not wanting to take part in the project may have perceived issues around the costs of taking part which meant they did not take part.

Second, due to the impact of the COVID‐19 pandemic, the intervention was conducted over the telephone and/or video‐conferencing (e.g. Attend Anywhere, Microsoft Teams) for seven participants. This could limit the findings of the current study, as we were unable to evaluate the acceptability of a full face‐to‐face intervention for a number of participants. However, we can offer a nuanced perspective on the acceptability of different modes of CBSPp which could provide a basis for further research into the efficacy of remote CBSPp. Furthermore, participants did not feel that receiving therapy over the telephone affected the quality, acceptability, or effectiveness of CBSPp. They reported better perceived anonymity (e.g., feeling embarrassed about showing their emotions to the therapist) and increased accessibility (Bee et al., 2016; Turner et al., 2018). This is important for people who cannot attend face‐to‐face appointments due to personal circumstances or health, financial, time, and logistical challenges (Cavanagh et al., 2018; Mohr et al., 2010). Delivery of therapy remotely to meet growing public demands is increasing (Rushton et al., 2020). Therefore, it is important to understand the impact of the pandemic on remote service provision. Recent studies have shown that remote therapy can be a trustworthy and feasible alternative to face‐to‐face therapy (Murphy et al., 2020; Rushton et al., 2020; Stefan et al., 2021; Tang et al., 2021). A qualitative study by Rushton et al. (2020) exploring people's views on telephone psychological interventions found that, despite initial reservations about treatment efficacy and anticipated communication barriers, on reflection, people reported a positive effect at the end of therapy, suggesting that telephone therapy can be effective. These results align with the experiences of some of our participants who had face‐to‐face and telephone therapy and did not feel that telephone intervention was inferior to face‐to‐face therapy.

### Recommendations for research and clinical practice

It would be useful to learn about therapists' perceptions and experiences of providing CBSPp during the pandemic and using different modes of delivery. Therapists may have certain reservations and expectations about delivering therapy remotely (Humer et al., 2020; McBeath et al., 2020). For example, being unable to meet with the client in person can impact the quality of the therapeutic alliance (Connolly et al., 2020; Ertelt et al., 2011). Moreover, disclosing sensitive information and managing risk issues during remote therapy may be daunting and stressful for therapists and clients alike. Therefore, adopting a fully flexible approach to therapy delivery that offers different modes, frequencies and durations, is essential for therapy inclusivity and acceptability. The next step in the investigation of the acceptability of CBSPp is to include therapists' views and experiences of delivery using the TFA. This type of investigation is currently missing in the literature.

A key finding was the importance of the alliance between clients and therapists. A stronger alliance has a positive effect on therapy outcomes for people experiencing psychosis and/or suicidality (Browne et al., 2019). Awareness and management of the sensitivity around the potential of therapy to make people feel worse if they felt unable to complete tasks are paramount for ensuring alliance and therapy acceptability. Some participants felt that the therapy resources were too complex and overwhelming which made them difficult to refer to. Therefore, resources can be simplified in a way that would be helpful for people to use with the view to increasing therapy acceptability and effectiveness. For example, it would be useful to include specific guidance on how to implement the staying well plan following cessation of therapy. This may help participants feel better prepared to use the plan when needed.

Clients' understanding of the nature and aims of an intervention is essential for ethical therapy research (Mishara & Weisstub, 2005). Our findings highlight the importance of providing clear information about the essence of CBSPp, since the understanding of what constitutes suicidal experiences can vary and suggest amendments to the type of information given to potential participants. It is important to understand discrepancies between people's anticipated and experienced acceptability or effectiveness of an intervention prior to and after receiving therapy.

## CONCLUSION

The TFA provided a useful framework to understand people's experiences of CBSPp. Participants described a range of ways in which CBSPp was perceived to be acceptable and beneficial. Talking about suicide was difficult but with a trusting relationship with therapists, it was perceived to be useful for understanding psychosis and suicidal experiences. It is important for therapists to ensure that clients' understanding of the CBSPp's aims is clear and aligns with their expectations of effectiveness, and to invest in building strong therapeutic alliances. Offering flexible modes of CBSPp delivery can enhance intervention acceptability and engagement. Future research will benefit from examining therapists' experiences and perceptions of delivering CBSPp.

## AUTHOR CONTRIBUTIONS

SP, YA, PG and GH designed this qualitative study with input from grant holders on the CARMS study (Gooding et al., 2020) and experts by experience (the CARMers). Data were collected by LC, Paul Marshall (PM), KH, and YA. KH and LC led the data analysis and drafted the manuscript, supervised by SP. SP, EP, LC, and KH contributed to data analysis and writing of the paper. Analysis was supported by discussions from the CARMS operational team, in particular GH, DP, YA, and PG. All authors read and approved the final manuscript.

## CONFLICT OF INTEREST STATEMENT

All authors were principal investigators and co‐investigators in the trial. Some of the co‐authors were employed as research assistants or project coordinators on the trial.

## ETHICAL APPROVAL

This study has been reviewed and approved by the NHS HRA (ref: 201644).

## Supporting information


Data S1.

## Data Availability

The raw data are not publicly available due to privacy or ethical restrictions (see https://authorservices.wiley.com/author‐resources/Journal‐Authors/open‐access/data‐sharing‐citation/data‐sharing‐policy.html).
