# Supplementary material for: Acceptability of a novel suicide prevention psychological therapy for people who experience non‐affective psychosis
Source: Psychol Psychother. 2023 Mar 1;96(3):560–76. doi: 10.1111/papt.12456 (PMC10953419; doi:10.1111/papt.12456)
Supplement: Supplementary file 1 — Data S1. [file PAPT-96-560-s001.docx]

**Topic Guide**

**CARMS Qualitative Study Patient Feedback of Therapy (Updated Feb 2018)**

*What follows is a guide. The order and exact content of the questions will be determined by the participant and will be influenced by the ongoing analysis so the order of the questions may vary as the interview develops.*

*The following topics and prompts serve as an interview guide.*

*Probe and ask for examples as the time permits.*

**Introductions**

- Introduce self, thank participant for attending
- Explain purpose of interview stressing interested in both positive and negative views and experiences.
- Reiterate that we do not work directly with the therapists so anything that the participant shares will not go back to the therapists.
- Clarify whether participant is currently experiencing COVID-19 symptoms and if they are self-isolating.
- Check participant is in a quiet room where they are unlikely to be disturbed.

**Background information: Participants situation prior to CARMS therapy**

- Can you tell me a little about what was going on for you before you started therapy?
  - PROBES**:** How was your mental health at that time?
  - What sort of problems were you experiencing?
  - What treatments or help were you receiving? What were your views about these?
  - How has your therapy changed since the COVID-19 outbreak? Eg. is participant now receiving therapy over the phone?

**Views / prior experiences of psychological therapy / ‘talking treatments’**

- Have you ever had any type of psychological therapy before the CARMS therapy?

**If Yes:** What kind of therapy was that?

PROBES: CBT Cognitive Behavioural Therapy / DBT Dialectical Behavioural / Therapy / EMDR / Counselling / Cognitive Restructuring Therapy / Other?

Was the therapy: in a Group / Individual 1:1 / Telephone / Online / Family Therapy?

Who delivered the psychological therapy? PROBE: Clinical Psychologist / CBT Therapist/ IAPT, Nurse / Psychiatrist / Counsellor / Other?

- What did you think about it? How did it help or not help? What effect did it have on your suicidal feelings?

- **If No :** Were you offered psychological therapy? Would you have liked to have tried psychological therapy?
- **Experiences of suicidality prior to CARMS therapy:**
- When did you start feeling suicidal? How long had that been going on?
- How bad were you feeling – how often did you think about suicide? Were there times when you harmed yourself? Can you give me an example of that?
- How did you cope when feeling suicidal? What helped? What made things worse?
- How easy or difficult did you find it to talk about your suicidal feelings?

**Experiences of CARMS therapy for suicidality**

**Expectations**

- What were your views about being offered a psychological therapy particularly designed to help with suicidal thoughts and behaviour? What concerns if any, did you have?
- How did you expect therapy would be before you started? What did you want from therapy?
- How did you feel about the move to phone therapy? How has your experience of phone therapy compared to your experience of face to face therapy? Is one preferable to the other? If yes, why?

**Views of impact of therapy**

- For you, what was the most important thing about CARMS therapy?
- What were the most difficult or unhelpful aspects of therapy?
- What was the most useful thing you learnt from therapy?
- Has your life changed in any way since having CARMS therapy?

PROBES: relationships / work / meaningful activity / social life / recreational activities / physical health / general mental health / other treatments used (use of medications / alcohol/ street drugs etc?

- What effect, if any, did CARMS therapy have on your experience of suicidal thoughts and behaviour?

PROBES: Any changes in frequency / strength / intensity of thoughts?

Any changes in frequency or severity of suicide attempts / self-harm behaviour?

- Can you describe any times when you felt therapy worsened your suicidal thoughts?
- Can you tell me about any suicidal attempts that you made when you were receiving CARMS therapy?
- Can you tell me about any suicidal attempt or self-harm behaviour since finishing CARMS therapy?
- Do you still get suicidal thoughts now?
- **If YES:** How do you manage these on-going suicidal thoughts or feelings?
- **If NOT:** What effect has this had on your life?

**Views of how therapy integrated with general mental health care**

- When you were having therapy did your usual mental health workers ask you about the therapy?
- What were their views about you having CARMS therapy?
- How much or little did CARMS therapy fit in with your general mental healthcare / treatments? PROBES: did you see it as being complimentary or very stand-alone and different to your usual treatment?

**Views about duration & ending of CARMS therapy**

- How many therapy sessions did you have? PROBES: Were you satisfied with that number? Were the sessions too long or too short?
- What were your views as the therapy was coming to an end? PROBES: Did you have any concerns? Can you tell me about these? Did you have any discussions with the therapist about preparing for the end of therapy? What ideally would you like to have happened?

**Continuing use of CARMS techniques following end of therapy**

- What aspects of CARMS therapy do you still use now? Why / when do you practice that?
- What effect does that have for you?

**Views about therapist**

- What were your expectations of the therapist?
- What did you think his / her role would be?
- What was most important to you about working with the therapist?
- How would you describe your relationship with the therapist?
- How was your relationship with the therapist over the phone? Could you describe any aspects that were positive/negative?

**Views about homework**

- What were your views about the “homework” part of therapy? Why do you think the homework was given?
- Were you able to do the homework?
- What effect did the homework have for you? If not completed enquire why?

**Endings**

Identify whether participant wishes to offer any further information about anything?

**Thank the participant.**
